# Individual risk assessment tool for school‐age asthma prediction in UK birth cohort

**DOI:** 10.1111/cea.13319

**Published:** 2019-01-04

**Authors:** Ran Wang, Angela Simpson, Adnan Custovic, Phil Foden, Danielle Belgrave, Clare S. Murray

**Affiliations:** ^1^ Division of Infection Immunity and Respiratory Medicine School of Biological Sciences Manchester Academic Health Science Centre Manchester University NHS Foundation Trust The University of Manchester Manchester UK; ^2^ Department of Medicine Section of Paediatrics Imperial College London London UK

## Abstract

**Background:**

Current published asthma predictive tools have moderate positive likelihood ratios (+LR) but high negative likelihood ratios (−LR) based on their recommended cut‐offs, which limit their clinical usefulness.

**Objective:**

To develop a simple clinically applicable asthma prediction tool within a population‐based birth cohort.

**Method:**

Children from the Manchester Asthma and Allergy Study (MAAS) attended follow‐up at ages 3, 8 and 11 years. Data on preschool wheeze were extracted from primary‐care records. Parents completed validated respiratory questionnaires. Children were skin prick tested (SPT). Asthma at 8/11 years (school‐age) was defined as parentally reported (a) physician‐diagnosed asthma and wheeze in the previous 12 months *or* (b) ≥3 wheeze attacks in the previous 12 months. An asthma prediction tool (MAAS APT) was developed using logistic regression of characteristics at age 3 years to predict school‐age asthma.

**Results:**

Of 336 children with physician‐confirmed wheeze by age 3 years, 117(35%) had school‐age asthma. Logistic regression selected 5 significant risk factors which formed the basis of the MAAS APT: wheeze after exercise; wheeze causing breathlessness; cough on exertion; current eczema and SPT sensitisation(maximum score 5). A total of 281(84%) children had complete data at age 3 years and were used to test the MAAS APT. Children scoring ≥3 were at high risk of having asthma at school‐age (PPV > 75%; +LR 6.3, −LR 0.6), whereas children who had a score of 0 had very low risk(PPV 9.3%; LR 0.2).

**Conclusion:**

MAAS APT is a simple asthma prediction tool which could easily be applied in clinical and research settings.

## 1 INTRODUCTION

Preschool wheeze is prevalent^1^, ^2^ and incorporates several different phenotypes, with varying prognosis.^3^, ^4^, ^5^, ^6^, ^7^, ^8^ In some children it is transient; in others it may indicate early signs of persistent asthma. In clinical practice, the identification of the children with preschool wheeze who are at high risk of developing asthma in later childhood may aid parents and facilitate clinicians in early risk stratification, allowing closer follow‐up for those at risk.

Asthma predictive tools have been developed previously,^9^, ^10^, ^11^, ^12^, ^13^ and likelihood ratios (LR) have been frequently used to assess and validate the predictabilities of these tools.^10^, ^14^, ^15^, ^16^ High positive LRs (+LR) “rule‐in” the disease when the test is positive, while low negative LRs (−LR) “rule‐out” the disease when the test is negative.^17^, ^18^ For a good predictive tool, a high +LR and a low −LR are required.^14^ A +LR of >10 and a −LR of <0.1 are likely to have substantial impact on clinical decision making.^19^ In contrast, a +LR between 1 and 2 and a −LR between 0.5 and 1 alter disease probability by a small and less clinically significant degree.^19^ The ease of application and consistency of performance of a predictive tool also determines its clinical applicability.

The Asthma Predictive Index (API) from the Tucson Children's Respiratory Study^9^ was the first of several published predictive tools. The API includes invasive investigations (blood eosinophil levels) and has a moderate +LR (4.9 for asthma at age 8) with a high −LR (0.9), making it a moderate prediction tool for “ruling in” school‐age asthma but not helpful in “ruling out”.^14^ Validation studies have expressed reservations about its ease of implementation and clinical usefulness.^14^, ^20^, ^21^ Subsequently, other studies have developed predictive tools.^10^, ^11^, ^12^, ^22^ However, these often achieve moderately high +LRs at a cost of high −LRs.^14^ Some included predictors solely from clinical history to improve ease of use in clinical settings.^10^, ^12^ However, the additions of minimally invasive objective predictors (eg, skin prick tests [SPT]) may add additional value to the predictive tool.

In this study, amongst children who have documented wheeze in their primary‐care records within the first 3 years of life, we aimed to use predictors from clinical history and SPTs collected at age 3 years to develop a clinically useful risk stratification tool for asthma in the school‐age.

## 2 METHODS

### 2.1 Study design, setting and participants

We analysed data from the Manchester Asthma and Allergy Study (MAAS). This is a population‐based birth cohort described in detail elsewhere.^23^ In brief, parents were screened for eligibility at antenatal clinics. The study protocol was approved by the Local Research Ethics Committee, (South Manchester ERP/94/032, ERP/95/137, 03/SM/400, 06/Q1403/142) and all parents gave written informed consent. We used data collected at follow‐up at ages 3, 8 and 11 years for this analysis. Validated questionnaires were interviewer‐administered to collect information on parentally reported symptoms^24^; the questions used from the validated questionnaire are presented in Table S1 in the Online Repository. Allergic sensitization was ascertained using skin prick tests (SPT) at the age of 3. A trained paediatrician extracted data from primary‐care medical records.^25^ Children with GP‐confirmed wheeze with complete data set for the predictor variables at the age of 3 years, and available data to define asthma at school‐age (the clinical outcome variable) at age 8 or age 11 years, were included in the analysis.

### 2.2 Definition of variables

#### 2.2.1 Primary‐care physician (general practitioner—GP) confirmed wheeze by age 3 years

The presence of wheeze documented in primary‐care record by age 3 years.

#### 2.2.2 Asthma at school‐age (at age eight and/or age 11 years)

Parentally reported, either (a) physician‐diagnosed asthma and wheeze in the previous 12 months or (b) more than 3 wheeze attacks in the previous 12 months.

#### 2.2.3 Current eczema (age 3 years)

Parentally reported, answered positively to both “Did the doctor ever tell you that your child had eczema?” and “Does your child still have eczema?”

#### 2.2.4 Allergic sensitisation (age 3 years)

Skin prick tested mean weal diameter at least 3 mm greater than the negative control to any of the allergens tested (house dust mite, cat, dog, grasses, moulds, milk and egg [Bayer, Elkahrt, IN, USA]).

### 2.3 Statistical analysis

Relevant variables were assessed in univariate logistic regression models and these variables were entered into a backward stepwise multivariable logistic regression model. Predictors that improved the model fit (Akaike and Bayesian Information Criterion, AIC and BIC) were included in MAAS APT. A forward stepwise multivariable logistic regression model was used to assess the robustness of the backward stepwise selected model. Any differences in the variables selected were resolved using the discriminative ability of the model, model fit (AIC, BIC) and clinical interpretation of the results. We derived a simple scoring system by rounding up regression coefficient to the nearest integer. The total score was calculated for each subject. We assessed the discriminative ability of this model using a receiver operating characteristic (ROC) curve and the area under the curve (AUC). A multiple imputation model was developed to deal with missing data, and the model was internally validated (See online repository for details). Statistical significance throughout the manuscript was at the 1% significance level to account for multiple testing. Analyses were performed using SPSS 20 (IBM, New York, USA), STATA 13 (StataCorp, Texas, USA) and R version 3.3.1.

## 3 RESULTS

### 3.1 Study population

Out of 1184 participating families recruited during pregnancy, 995 children completed follow‐up at age 3 years, and 916 (92%) had primary health care records data extracted. Of these, 336 children had documented and confirmed wheeze in their health care record by age 3 years. These children were included in the model development (Figure S1). Asthma at school‐age (8 or 11 years) was present in 117/336 (35%) children.

### 3.2 Development of the model

Of the 22 predictors measured at age 3 years which were assessed in the univariate logistic regression analysis, 11 were significantly associated with asthma at school‐age (Table S2. *P* ≤ 0.01). When the predictors were entered in a multivariable logistic regression model (using a backward stepwise selection procedure), five remained in the model: (a) Wheeze after exercise; (b) Wheeze causing shortness of breath; (c) Cough on exertion; (d) Current eczema; and (d) Allergic sensitisation (Table 1). When a forward stepwise selection procedure was performed on the 22 predictors, the same five predictors were selected. Combining these predictors into an asthma predictive tool (MAAS APT, Table 1) allows children to score a minimum of zero, up to a maximum of five points.

**Table 1:** Backward stepwise multivariable logistic regression of predictors recorded at age 3 years, for possibility of developing asthma at school‐age

	Adjusted ORs (*P*‐values)	RC	Bootstrapping [RC 95%CI]	Bias^a^	Simplified RC
Demographic and perinatal data					
Gender	1.082 (0.820)	‐	‐	‐	‐
Paternal asthma ever	1.363 (0.474)	‐	‐	‐	‐
Maternal asthma ever	0.802 (0.557)	‐	‐	‐	‐
Paternal smoking at recruitment	1.037 (0.940)	‐	‐	‐	‐
Maternal smoking at recruitment	0.956 (0.968)	‐	‐	‐	‐
Paternal smoking age 3	0.829 (0.645)	‐	‐	‐	‐
Maternal smoking at age 3	1.403 (0.408)	‐	‐	‐	‐
Parental atopy (at least one parent)	0.993 (0.989)	‐	‐	‐	‐
Wheeze‐related symptoms at age 3					
Wheeze require meds	1.309 (0. 496)	‐	‐	‐	‐
Wheeze without cold	1.739 (0.136)	‐	‐	‐	‐
Wheeze with cold air	1.062 (0.917)	‐	‐	‐	‐
Wheeze after exercise	2.679 (0.018)	0.985	[0.146‐1.899]	0.026	1
Wheeze causes SOB	2.343 (0.007)	0.851	[0.255‐1.525]	0.020	1
Wheeze attack of more than three times	1.269 (0.625)	**‐**	**‐**	**‐**	**‐**
Atopic status					
Current eczema	2.627 (0.002)	0.966	[0.354‐1.634]	0.021	1
SPT sensitisation	3.342 (<0.001)	1.207	[0.549‐1.887]	0.012	1
Physician‐diagnosed hayfever/allergic rhinitis	0.918 (0.885)	‐	**‐**	**‐**	**‐**
Cough‐related symptoms at age 3					
Cough mainly at night	0.897 (0.736)	‐	‐	‐	‐
Cough when excited	1.000 (1.000)	‐	‐	‐	‐
Congestion/phlegm apart from colds	3.091 (0.079)	‐	‐	‐	‐
Cough on exertion	2.972 (0.001)	1.089	[0.414‐1.895]	0.047	1
Cough with cold air	1.238 (0.603)	‐	‐	‐	‐

The MAAS APT to be applied at age 3 years to predict asthma at school‐age	
	Responses:
✓ Do the wheeze attacks cause shortness of breath? (1)	Y□ N□
✓ Does the child wheeze after exercise? (1)	Y□ N□
✓ Does the child cough after exertion? (1)	Y□ N□
✓ Does the child have eczema currently? (1)	Y□ N□
✓ Is the child skin prick test positive to common allergens? (1)	Y□ N□
Total score	__/5
0 ‐ low risk	□
1‐2 ‐ Medium/indeterminate risk	□
≥3 ‐ High Risk	□

RC, Regression coefficient.

a Bias: difference between the average value of RC across the bootstrap samples and RC in original sample.

**Figure 1:**
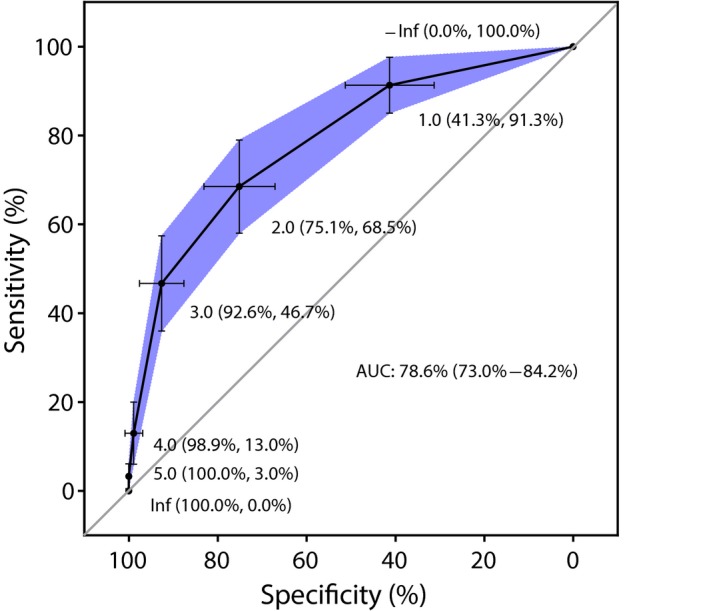
ROC Curve of MAAS APT at age 3 in predicting school‐age asthma

A total of 281 (84%) children had complete data available on all 5 predictors at age 3 years and were used to test the MAAS APT. Of the 281, 92 had asthma at school‐age. The risk of asthma at school‐age increased with increasing MAAS APT score (Table 2). The model showed good discriminative ability (AUC = 0.79, Figure 1). A cut‐off score of ≥1 gave a low −LR (0.2) (sensitivity 91%, specificity 41%, PPV 43% and NPV 91%, +LR = 1.6) and a cut‐off score of ≥4 gave a high +LR (12) (sensitivity 13%, specificity 99%, PPV 86% and NPV 70%, −LR = 0.9) (Table 3).

**Table 2:** Risk of asthma at school‐age with each score derived at the age of 3

MAAS APT score Number N=281	Number of children within each score developing school‐age asthma (% within the score, row%)	% of children with asthma who had each score (n=92, column %)
Score 0 n=86	8 (9.3%)	8.6%
Score 1 n=85	21 (24.7%)	22.8%
Score 2 n=53	20 (37.7%)	21.7%
Score 3 n=43	31 (72.1%)	33.7%
Score 4 n=11	9 (81.8%)	9.8%
Score 5 n=3	3 (100%)	3.2%

**Table 3:** Performance measures of MAAS APT for different cut‐off scores

Score cut‐off	Sensitivity	Specificity	PPV	NPV	+LR	−LR
≥1	91	41	43	91	1.6	0.2
≥2	68	75	57	83	2.8	0.4
≥3	47	93	75	78	6.3	0.6
≥4	13	99	86	70	12	0.9
5	3	100	100	68	∞	1.0

+LR calculated as sensitivity/(1‐specificity); −LR calculated as (1‐sensitivity)/specificity.

Of those who scored 0 (n = 86), only 9.3% had asthma at school‐age, (OR: 0.14 [95%CI 0.06‐0.3], *P* < 0.001); only 8.7% of children who had asthma at school‐age had a 0 MAAS APT risk score at age 3 years (Table S3). A MAAS APT score of 0 gave a low −LR (0.2). Therefore, we defined a MAAS APT score of 0 as “Low risk” (Table 1).

For the children who scored 5 on the MAAS APT, all three had asthma (100% risk, Table 2). Almost half of children with school‐age asthma had a score of ≥3 at the age of 3, with a false positive rate of only 7.4% (Table S3). Children with a score ≥3 had a greater than 75% risk of school‐age asthma (+LR 6.3, −LR 0.6; OR: 11.0, [95%CI 5.6‐21.7], *P* < 0.001). Therefore, we defined a MAAS APT score ≥3 as “High risk” (Table 1).

Almost half of the children had a score of 1 or 2, and the risk of asthma at school‐age was difficult to predict in these children (+LR 0.9, −LR 1.1) Of the 138 children that fell within this group, 29.7% had asthma at school‐age, accounting for 45% of the asthma cases (Tables 2 and S3). Therefore, we defined a MAAS APT score of 1‐2 as indeterminate risk.

### 3.3 Multiple imputation and internal validation

Fifty data sets, each with 336 patients were created. The regression coefficients (Table S4) from the final model selected in the original complete cases analysis, calculated from the pooled analysis of the 50 imputed data sets were similar to those from the model in the original data set. The calibration values (Table S5) indicate the degree of agreement between observed outcomes and predictions.

## 4 DISCUSSION

### 4.1 Summary of results

We developed a simple prediction tool for asthma at school‐age amongst children who had physician‐confirmed wheeze in the first 3 years of life, within the context of a population‐based birth cohort. In developing the MAAS predictive tool, we assessed severity, frequency and triggers of wheeze and cough, other atopic history, family history, smoking exposure, atopic status and demographic details. This final model comprises only 5 predictors, resulting in a score between 0 and 5. The tool provides moderately high +LR (6.3) and a reasonably low −LR (0.6) in children with a high score (≥3, ~20% of all wheezy children at the age of three). Conversely, for children who had confirmed wheeze, but without the presence of any predictive features stated in MAAS APT (score = 0), the risk of asthma at school‐age was very low (<10%; LR 0.2).Therefore, for children with GP‐confirmed wheeze by age 3 years, the MAAS APT score performs well for those with high scores and those with low scores.

Amongst children with confirmed wheeze at age 3 years, half had a MAAS APT score of 1 or 2; and because of this poor discriminative ability, we termed this an “indeterminate” risk group. This large “indeterminate” group accounted for almost half (45%) of the children who developed school‐age asthma. We speculate that within this “indeterminate” group, ongoing and future environmental factors may determine the persistence of wheeze in these children who had wheezed in early life. Furthermore, it is possible that in this “indeterminate” group of children, development of school‐age asthma is potentially preventable with an environmental modification, and future studies of secondary prevention may wish to target this group.

### 4.2 Comparison with previous prediction models

Since the development of API,^9^ studies have been carried out to validate its reliability in South America^21^ and UK populations.^20^ These results were comparable to the original paper. However, it is recognized that due to its moderate positive likelihood ratio but high negative likelihood ratio, the applicability in the clinical setting is limited.^14^, ^20^, ^21^ Subsequently, a number of other studies have tried to develop a tool that is more discriminative than the API.

The PIAMA risk score developed from a Dutch birth cohort was based on 8 predictors.^12^ The AUC (0.74) was similar to that of MAAS APT, but PPV in those with a high score was only 50% and the sensitivity was extremely low (7%).^12^ External validation of PIAMA risk score showed good agreement, and a modified score was developed.^15^ However, the high‐risk group had lower sensitivity (11.6%) and PPV (26.4%) compared to that of MAAS APT (47% and 75%, respectively).^15^ A Norwegian group developed a predictive scoring system to predict asthma at the age of 10 years based on the severity of obstructive airway disease at the age of 2.^13^ However, the suggested cut‐off value by the authors gave a PPV of only 54.3% with moderate +LR (4.3) and −LR (0.6). A UK‐based study also developed a score to predict persistence of wheeze at age 10 years in those that wheezed in the first 4 years of life.^11^ This score included 4 factors collected at different time‐points in early life (age 1, 3 and 4 years). A maximum score (4), using this tool, had a moderately high +LR (7.9) for persistence of wheeze, but the negative likelihood ratio was also high (0.9). In addition, the need of obtaining clinical information at age 1, 3 and 4 years also markedly reduces its ease of use and clinical applicability.

Another UK‐based study of 1226 children with clinically significant wheeze or cough at age 1‐3 years who presented to the primary‐care physician were analysed in the Leicestershire Respiratory Cohort 5 years later,^10^ and an asthma prediction tool was developed. The tool consists of 10 questionnaire based predictors. In addition, the development of the tool was solely based on parentally reported symptoms, and no extraction of GP records was carried out. The discriminative ability in terms of AUC was comparable to the MAAS APT. The high‐risk group gave a higher +LR than MAAS APT (9.4 vs 6.3, equivalent to ~5% difference in clinical probability^14^), but a much lower sensitivity (22% vs 47%) and a higher −LR (0.8 vs 0.6). The tool simply involves administration of a questionnaire with 10 questions. However, some questions use rather subjective terms including “a little” and “a lot.” In comparison, MAAS APT is designed to be succinct for clinical applicability and includes 5 yes/no questions. This avoids excessive subjective measurement and potential inter‐observer discrepancies or reporting bias. The addition of SPT to determine sensitisation contributes to the predictive ability of the tool. In our cohort, the addition of SPT in MAAS APT identified a further 19 children who were at high risk of school‐age asthma, compared to MAAS APT without SPT as a predictor. Of these 19 children, 14 had asthma at school‐age. This accounts for 33% of all children who had school‐age asthma who were in high‐risk group.

It is also of interest that in this cohort parental history of asthma/atopy (ie, heritable components) was not statistically significant in the prediction of school‐age asthma.

Although MAAS APT showed slightly better predictive abilities reflected in AUC, +LR and −LR when compared to previously developed asthma predictive tools, the increase in the risk of school‐age asthma is only moderate for clinical interpretation, and the risk of misclassification remains significant. A proposed simplification for interpretation of likelihood ratios into clinical probability was previously published.^16^ According to this, a +LR of 6.3 in high‐risk group (score ≥3) corresponds to increased probability of school‐age asthma of approximately 35% and a +LR of 0.2 in the low‐risk group (score = 0) corresponds to a decreased probability of asthma of about 30%.

### 4.3 Strengths and limitations

Unlike many of the established asthma prediction tools, MAAS APT was developed within a cohort of children who had GP‐confirmed diagnosis of wheeze. In the current cohort, 28% (94/336) of children who had GP‐confirmed wheeze, on questioning the parents denied that the child had ever wheezed by the age of 3 years. The poor correlation between parentally reported wheeze and physician‐confirmed wheeze is well recognized.^26^, ^27^ Given the discrepancies between parentally reported and physician‐confirmed wheeze, a reliable and clinically useful asthma prediction tool should probably only be developed from a cohort of children who have had physician‐confirmed wheeze.

The MAAS APT is developed from a representative cohort of children, where the tools would be potentially applied. Although the number of children from which the tool was developed is small compared to other studies, the development method is robust, and a multiple imputation model was developed to account for potential bias caused by missing data. This multiple imputation method assumes that the missing data are missing at random (MAR). This means that the probability that data are missing does not depend on unobserved data but may depend on observed data. Conditioning on the observed data that is related to missingness would mean that the remaining missingness is completely at random and, therefore, ignorable. The MAR assumption cannot be tested statistically; the plausibility of the MAR assumption and the variables chosen as related to missingness should be considered carefully. In this data set, we believe the MAR assumption is reasonable and the variables identified in the comparison between those with and without complete data were used in the imputation model.

To make the current method more robust and minimize “overfitting,” optimal estimates and 95% CIs from the backward stepwise logistic regression model were obtained by using the bootstrapping technique. This technique was also used to internally validate the prediction model, including the model selection process. This method of internal validation may be better suited over other methods of cross‐validation with the given sample size.^28^, ^29^ This method “fine‐tunes” pre‐selected predictors and develops a highly simplified model. Variables, where a small number of patients are in one category, may not be accurately reflected in the bootstrapping procedure since a simulated data set may result in such a variable having no patients in a particular category. However, MAAS APT has not yet been validated in an external cohort.

We recognize that SPTs may not be readily available in primary care. However, skin tests are available in most secondary care settings, are less invasive than serum eosinophil counts, and our data show that they add value to the MAAS APT.

## 5 CONCLUSION

MAAS APT may be a useful tool which combines clinical history and objective measures in predicting future risk of asthma in both clinical and research settings. It provides a simple assessment for asthma risk at school‐age in young children. Although internally validated, further external validation of this tool is needed.
