# Supplementary material for: Individual risk assessment tool for school‐age asthma prediction in UK birth cohort
Source: Clin Exp Allergy. 2019 Jan 4;49(3):292–8. doi: 10.1111/cea.13319 (PMC6446726; doi:10.1111/cea.13319)
Supplement: Supplementary file 1 [file CEA-49-292-s001.docx]

**INDIVIDUAL RISK ASSESSMENT TOOL FOR ASTHMA PREDICTION AT SCHOOL AGE AND IN UK BIRTH COHORT - ONLINE REPOSITORY**

**METHODS**

**Table E1 VARIABLE DEFINITIONS**

| **Variables** | **Predictor Definitions** |
| --- | --- |
| ***Wheeze*** | |
| GP recorded wheeze at age 3 | A trained paediatrician extracted data from primary care medical records: defined as presences of wheeze on GP record up to the age of 3. |
| Wheeze requires medication | “Has your child ever required medicine or treatment for the(se) attack(s)?” |
| Wheeze without cold | “Does your child’s chest wheeze or whistle when occasionally apart from with colds?” |
| Wheeze with cold air | “Does your child wheeze when exposed to cold air?” |
| Wheeze after exercise | “Does your child ever get attacks of wheezing after he/she has been playing or exercising?” |
| Wheeze causes SOB: | “ Has your child ever had an attack of wheezing that has caused him/her to be short of breath?” |
| Wheeze attack of more than 3 times | “ Has your child had three or more episodes of attacks of wheezing that caused him/her to be short of breath?” |
| ***Atopic status at age 3*** | |
| Current eczema | Answer “yes” to both “did the doctor ever tell you that your child had eczema?” and “does your child still have eczema?” |
| Physician diagnosed rhinitis | “Has your doctor ever told you that your child has hay fever/allergic rhinitis?” |
| ***Cough*** | |
| Cough mainly at night | **“**Does your child cough mainly at night?” |
| Cough excited**:** | “Does your child cough when he/she is excited?” |
| Cough on exertion**:** | “Does your child cough after exertion?” |
| Cough with cold air | **“**Does your child cough on exposure to cold air?” |
| Congestion/phlegm apart from colds**:** | **“**Does your child usually seem congested in the chest or bring up phlegm apart from colds?” |

**MULTIPLE IMPUTATION AND INTERNAL VALIDATION**

A multiple imputation model was used to create 50 datasets, each with 336 patients (number of children with GP recorded wheeze at the age of 3 years and outcome variable of school age asthma). Table E5 shows the results of the final model, selected in the original complete cases analysis, calculated from the pooled analysis of the 50 imputed datasets. The pooling of the results of the analysis from the 50 datasets used Rubin’s combination rules^1^.

The multiple imputation method used was chained equations. This was implemented in STATA 13 using the ‘mi impute chained’ command. The chained equations technique is an iterative sequence of univariate (one variable at a time) imputation methods with fully conditional specification (FCS) of prediction equations. In this study, each variable was imputed using all other variables considered for the imputation (excluding the variable itself).

All candidate variables and school age asthma, the outcome variable, were included in the multiple imputation models. Only gender, maternal asthma and parental atopy amongst the candidate variables did not have any missing data in the 336 children.

In order to internally validate the model, bootstrapping was used to account for overoptimism, the exaggeration of the AUROC (area under the receiver operating characteristic curve) due to producing the model and testing it in the same dataset. This was done by re-sampling the data and then running the model selection stage to produce a final model; the AUROC of this model was compared in the bootstrapped dataset and the original dataset to calculate the overoptimism for that dataset. This was repeated 1000 times. The analysis was also repeated with each multiply imputed complete dataset so that bootstrapped datasets were taken from the multiply imputed datasets.

The calibration values in table E6 indicate the degree of agreement between observed outcomes and predictions. Calibration-in-the-large reflects whether the predictions are too high or too low (a value of 0 would be ideal) and the calibration slope reflects the degree of overfitting (if the value is smaller than 1, the ideal value).

*^1^Rubin, D. B. 1987. Multiple Imputation for Nonresponse in Surveys. New York: Wiley.*

**RESULTS**

**Figure E1. Population selection**

Total **n=1184** recruited during pregnancy

**n=995 -** completed questionnaire data at age 3 year FU visit

**n=916**

Primary care data extracted at age 3 years

Excluded

**n=336 had GP recorded wheeze**

(n=117 with school age asthma)

**n=456** had no evidence of wheeze on GP record

**n=22** did not attend age 8 and age 11 follow up

**n=17** did not attend age 8 follow up and were non-asthmatic at age 11 follow up

**n=85** did not attend age 11 follow up and were non-asthmatic at age 8 follow up

n=281 had full dataset at age 3 years to determine MAAS APT scoring

55 had missing data

**Table E2. Univariate logistic regression of 22 predictors at age 3 years of school-age asthma**

| n=336 | **ORs** | **95% CI** | **(*p*-values)** |
| --- | --- | --- | --- |
| ***Demographic and perinatal data*** |  | |  |
| Gender | 0.63 | 0.39-1.03 | 0.064 |
| Paternal asthma ever | 1.74 | 0.94-3.23 | 0.080 |
| Maternal asthma ever | 1.07 | 0.63-1.83 | 0.808 |
| Paternal smoking during pregnancy | 0.97 | 0.58-1.61 | 0.895 |
| Maternal smoking during pregnancy | 1.47 | 0.78-2.79 | 0.239 |
| Paternal smoking age 3 | 1.38 | 0.80-2.35 | 0.244 |
| Maternal smoking age 3 | 1.60 | 0.85-3.01 | 0.144 |
| Parental atopy (at least one parent)  ***Wheeze-related symptoms*** at age 3 | 2.42 | 1.08-5.42 | **0.032** |
| Wheeze require meds | 3.27 | 1.92-5.57 | **<0.001** |
| Wheeze without cold | 3.84 | 2.24-6.58 | **<0.001** |
| Wheeze with cold air | 3.95 | 1.83-8.51 | **<0.001** |
| Wheeze after exercise | 5.92 | 2.97-11.79 | **<0.001** |
| Wheeze causes breathlessness | 4.20 | 2.53-6.98 | **<0.001** |
| Wheeze attack of more than 3 times  ***Atopic status at age 3*** | 3.59 | 1.98-6.53 | **<0.001** |
| Current eczema | 2.84 | 1.72-4.70 | **<0.001** |
| Sensitised on SPT | 3.35 | 1.94-5.79 | **<0.001** |
| Physician diagnosed hay fever/allergic rhinitis | 2.58 | 1.05-6.29 | **0.038** |
| **Cough-related symptoms at age 3** |  |  |  |
|  |  |  |  |
| Cough mainly at night | 1.45 | 0.90-2.33 | 0.130 |
| Cough excited | 3.07 | 1.64-5.76 | **<0.001** |
| Cough on exertion | 4.23 | 2.50-7.18 | **<0.001** |
| Cough with cold air | 2.89 | 1.63-5.13 | **<0.001** |
| Congestion/phlegm apart from colds | 2.92 | 1.05-8.08 | **0.039** |

**Table E3**- **describes percentages of children who developed school age asthma in low (0), medium (1-2) and high (>3) APT risk groups based on clinical information collected at age 3.**

| **Risk groups at age 3**  **n (% of children)** | **Children who developed school-age asthma**  **n (% within children who developed school age asthma)**  **[n=92]** | **Children who did not develop school age asthma**  **n (% within those children who did not develop asthma at school age)**  **[n=189]** |
| --- | --- | --- |
| **Low risk n=86 (30.6)** | 8 (8.7) | 78 (41.3) |
| **Medium/indeterminate Risk**  **n=138 (49.1)** | 41 (44.6) | 97 (51.3) |
| **High Risk**  **n=57 (20.3)** | 43 (46.7) | 14 (7.4) |

**MULTIPLE IMPUTATION OF MISSING DATA AND INTERNAL VALIDATION**

**Table E4** **demonstrates the results of the final model, selected in the original complete cases analysis, calculated from the pooled analysis of the 50 imputed datasets**

| **Variable** | **RC** | **OR (95% CI)** | **p-value** |
| --- | --- | --- | --- |
| **Wheeze after exercise** | 1.05 | 2.85 (1.27-6.38) | 0.011 |
| **Wheeze causes SOB** | 1.00 | 2.71 (1.50-4.91) | 0.001 |
| **Current eczema (age 3)** | 0.82 | 2.27 (1.28-4.05) | 0.005 |
| **Sensitised SPT (age 3)** | 1.24 | 3.46 (1.83-6.54) | <0.001 |
| **Cough on exertion** | 1.11 | 3.03 (1.59-5.78) | 0.001 |

**Table E5** **demonstrates the degree of agreement between observed outcomes and predictions**

| **Dataset** | **Over optimism** | **Calibration-in-the-large** | **Calibration slope** |
| --- | --- | --- | --- |
| Original | 0.039 | 0.041 | 0.872 |
| Multiple imputation 1 | 0.036 | 0.041 | 0.885 |
| Multiple imputation 2 | 0.034 | 0.039 | 0.891 |
| Multiple imputation 3 | 0.034 | 0.038 | 0.894 |
| Multiple imputation 4 | 0.038 | 0.044 | 0.877 |
| Multiple imputation 5 | 0.039 | 0.044 | 0.876 |
| Multiple imputation 6 | 0.038 | 0.041 | 0.883 |
| Multiple imputation 7 | 0.034 | 0.037 | 0.898 |
| Multiple imputation 8 | 0.039 | 0.043 | 0.880 |
| Multiple imputation 9 | 0.036 | 0.041 | 0.883 |
| Multiple imputation 10 | 0.038 | 0.042 | 0.881 |
| Multiple imputation 11 | 0.032 | 0.034 | 0.903 |
| Multiple imputation 12 | 0.034 | 0.036 | 0.898 |
| Multiple imputation 13 | 0.034 | 0.034 | 0.902 |
| Multiple imputation 14 | 0.033 | 0.036 | 0.895 |
| Multiple imputation 15 | 0.036 | 0.041 | 0.886 |
| Multiple imputation 16 | 0.030 | 0.031 | 0.910 |
| Multiple imputation 17 | 0.037 | 0.043 | 0.878 |
| Multiple imputation 18 | 0.032 | 0.034 | 0.904 |
| Multiple imputation 19 | 0.033 | 0.034 | 0.903 |
| Multiple imputation 20 | 0.034 | 0.038 | 0.892 |
| Multiple imputation 21 | 0.031 | 0.035 | 0.900 |
| Multiple imputation 22 | 0.037 | 0.039 | 0.890 |
| Multiple imputation 23 | 0.037 | 0.041 | 0.883 |
| Multiple imputation 24 | 0.034 | 0.034 | 0.901 |
| Multiple imputation 25 | 0.039 | 0.041 | 0.882 |
| Multiple imputation 26 | 0.033 | 0.035 | 0.904 |
| Multiple imputation 27 | 0.037 | 0.040 | 0.886 |
| Multiple imputation 28 | 0.035 | 0.038 | 0.891 |
| Multiple imputation 29 | 0.037 | 0.041 | 0.884 |
| Multiple imputation 30 | 0.037 | 0.042 | 0.883 |
| Multiple imputation 31 | 0.036 | 0.038 | 0.891 |
| Multiple imputation 32 | 0.032 | 0.034 | 0.906 |
| Multiple imputation 33 | 0.037 | 0.042 | 0.883 |
| Multiple imputation 34 | 0.036 | 0.038 | 0.891 |
| Multiple imputation 35 | 0.034 | 0.038 | 0.894 |
| Multiple imputation 36 | 0.034 | 0.039 | 0.894 |
| Multiple imputation 37 | 0.036 | 0.038 | 0.891 |
| Multiple imputation 38 | 0.035 | 0.036 | 0.896 |
| Multiple imputation 39 | 0.034 | 0.038 | 0.890 |
| Multiple imputation 40 | 0.038 | 0.041 | 0.885 |
| Multiple imputation 41 | 0.033 | 0.037 | 0.895 |
| Multiple imputation 42 | 0.032 | 0.033 | 0.903 |
| Multiple imputation 43 | 0.035 | 0.037 | 0.899 |
| Multiple imputation 44 | 0.036 | 0.040 | 0.889 |
| Multiple imputation 45 | 0.034 | 0.034 | 0.900 |
| Multiple imputation 46 | 0.035 | 0.039 | 0.892 |
| Multiple imputation 47 | 0.036 | 0.038 | 0.893 |
| Multiple imputation 48 | 0.035 | 0.040 | 0.889 |
| Multiple imputation 49 | 0.034 | 0.036 | 0.897 |
| Multiple imputation 50 | 0.042 | 0.047 | 0.865 |

The overoptimism, calibration-in-the-large and calibration slope in the original dataset with missing data are 0.039, 0.041 and 0.872, respectively. The averages of these values in the multiply imputed datasets are 0.035, 0.038 and 0.891, respectively
